# “We Are More than Our Parents’ Mental Illness”: Narratives from Adult Children

**DOI:** 10.3390/ijerph16050839

**Published:** 2019-03-07

**Authors:** Pamela M. Patrick, Andrea E. Reupert, Louise A. McLean

**Affiliations:** Faculty of Education, 19 Ancora Imparo Way, Monash University, Clayton, VIC 3800, Australia; andrea.reupert@monash.edu (A.E.R.); louise.mclean@monash.edu (L.A.M.)

**Keywords:** intergenerational relationships, parental mental illness, adult children, parenting, relationships, interpretative phenomenological analysis

## Abstract

Although research on children of parents with mental illness is growing, few researchers have examined the long-term impact of parental mental illness on adult children. This study explored the potential impact of growing up with a parent with a mental illness on the parenting role assumed by adult children. The qualitative study included ten participants, who were individually interviewed using a semi-structured interview schedule. Interpretative phenomenological analysis (IPA) along with member checks were utilised to derive themes from participants’ narratives. Three main themes were identified, including: ‘this is me’, ‘a whole new world’, and ‘because of you’. ‘This is me’ consisted of narratives highlighting how adult children intentionally went about parenting in ways different from their parents, and ‘a whole new world’ captured the salient identity that parenthood served for adult children. The third theme, ‘because of you’ highlighted the challenges adult children faced in their parenting roles as a result of their childhood experience living with a parent with mental illness. Participants highlighted the main challenges to be an absence of a reference point and lack of informal social supports. Recommendations for mental health practitioners and future research are presented in order to develop better ways to support adult children and their families.

## 1. Introduction

Worldwide, it is estimated that one in six people will experience a mental health problem each week [1]. The 2013 Global Burden of Disease study cited depression as the most predominant mental health concern, followed by anxiety, schizophrenia, and bipolar disorder [1]. The World Health Organization (WHO) estimates that between 35% and 50% of people with severe mental health problems in developed countries and 76%–85% in developing countries receive no treatment [2]. Furthermore, many of these individuals with mental health concerns are likely to be parents themselves. Using population estimates, researchers reported that approximately 23.3% of children in Australia have at least one parent with a mental illness [3]. Though scarce, research suggests that certain types of mental illness may be associated with particular types of maltreatment. A national longitudinal study of over 7000 parents found that parental depression predicted an increase in physical abuse but not neglect. Conversely, a diagnosis of obsessive–compulsive disorder predicted neglect but not physical abuse [4]. Parental mental illness has also been shown to correlate with substance use disorders as well as the offspring’s substance use later in life [5]. Data from the National Survey of Child and Adolescent Well-Being found that when comorbidity (i.e., depression and substance use) was present in a parent, a significant relationship was present for neglectful parenting, physical abuse, and emotional maltreatment even after controlling for demographic variables [6]. 

Like their parents, children too, experience a range of different emotions and experiences as a result of parental mental illness. Themes commonly reported by children include experiencing a heightened sense of responsibility and worry as a result of wanting to do their part to support their parents. Furthermore, children described the unpredictability in the home environment and shame when their parent was behaving differently [7]. Additional risk factors include genetics, parental unavailability [8], and the modelling of emotional dysregulation [9]. As a result of their familial context, children of parents with mental illness were found to have high internalising (e.g., anxiety and depression) and externalising problems (e.g., aggression and oppositional behaviour) [10] as well as social isolation [11]. Parents with a mental illness have been shown to be less positive and more critical than parents who do not have a mental illness [12]. Collectively, these children are at increased risk of experiencing poorer social, physical, and psychological health compared to children in families not affected by parental mental illness [13,14], and, without protective factors in place, this risk may persist into adulthood [15]. 

Although the narrative surrounding parental mental illness has long been dominated by the risk discourse, more empirical research has shed light on possible protective or steeling factors that could play a positive role in children’s lives in spite of parental mental illness. For instance, Drost and colleagues [16] have shown that children of parents with mental illness find ways within their means to help their parents, which in turn provides them with a greater sense of control over their situation. Others have pointed out that the experience of caring for a relative with a mental illness may result in close family ties and promote independence [17]. Accurate knowledge about their parent’s mental illness has been identified as a protective factor for children, as it has been shown to facilitate resilience [18]. Other protective factors include high intelligence, adaptive problem-solving skills, social activities, self-understanding, and an ability to regulate emotions [19,20]. Environmental factors, including adequate housing and timely mental health support, also serve as protective factors for these families [17]. 

Increased awareness about the protective factors for children and their families has resulted in the development of various interventions, such as: ‘Paying Attention to Self (PATS)’, ‘Family Focus’ and ‘Let’s talk about children’ [18,21,22]. Although the surge in research and interventions are both encouraging and reassuring, these remain fairly recent developments that were not available to children decades ago. These children now belong to the ‘adult children of parents with mental illness’ category. Repeated research has shown the profound impact childhood experiences and familial relationships can exert on one’s mental health and social status in the long term [23,24]. Retrospective accounts from adults who experienced parental mental illness highlight common childhood narratives including stigma, uncertainty, and feeling unsafe, all of which are known to hinder protective factors, such as conversations about parental mental illness and help-seeking behaviours [25]. Some adult children described a strained relationship and regrets about a lack of connection with their parent [26]. Others reported difficulty forming an intimate connection with a significant other [23]. In comparison to adults in the general population, adult children of parents with mental illness were found to have lower self-esteem, increased psychosocial problems [27], and higher incarceration rates [28,29]. Consistent with these findings, almost two-thirds of adult children of parents with mental illness are reported to experience mental health concerns themselves along with a myriad of psychosocial difficulties [30]. 

Problems resulting from growing up with a parent with a mental illness can be intergenerational. Sometimes known as ‘toxic inheritances’, the process in which distortions, silences, and violence are passed down from one generation to the next can have serious intergenerational impacts in families experiencing parental mental illness [25]. From an intergenerational perspective, a growing body of research indicates that parents not only play a formative role in determining their children’s development but also have a significant influence on how their children will, in turn, parent their own children [31,32,33]. Until recently, the majority of intergenerational studies have concentrated on the extreme ends of parental dysfunction, including child abuse, parental separation [34,35] or the genetic transmission of mental illness [36,37]. Increased interest in intergenerational influences has led researchers to examine the effect, if any, of adult children’s experiences on the roles they assume with particular attention given to parenthood [25,38]. Although researchers [26,30,38] in this field have been instrumental in drawing attention to the need for further research and supports for adult children, the literature in this field is in its infancy. To date, much of the existing research focuses on the retrospective accounts of adult children who experienced parental mental illness as children [30,39,40]. However, more research is needed to understand how formative experiences impact on the subsequent roles assumed by adult children.

One of the few studies that explored the experiences of adult children in relation to their parenting roles provides some insight. Murphy and colleagues [38] conducted a qualitative study involving 13 adult children with no past or current mental health concerns. The study identified parenting worries as a salient theme for adult children. Participants attributed their worries to the lack of an internal parenting framework, which perpetuated parenting self-doubts. Taken together, existing research suggests that the offspring of parents with mental illness are more susceptible to mental illnesses due to genetic vulnerabilities. Additionally, having grown up with parental mental illness has shown to impact psychosocial outcomes and perpetuate adult children’s parental worries and anxieties.

The current study aimed to extend Murphy and colleagues’ study [38] by examining the experiences of adult children specifically in relation to their parenting roles. Although the current paper has a similar aim to that of Murphy and colleagues, it is novel in its inclusion of participants with past and/or current mental health concerns. Given substantial research on the heredity of mental illness [41] and increased prevalence of mental health concerns in offspring of parents with mental illness [42,43], it is just as crucial to include adult children who have experienced mental illnesses to gain a nuanced appreciation of their challenges and perspectives. A qualitative study is integral in this regard, particularly for areas of study that remain underdeveloped, as it allows for a rich understanding of a topic area.

## 2. Materials and Methods

### 2.1. Sampling and Participants

In the current paper, ‘parents with a mental illness’ included individuals affected by at least one psychiatric disability (i.e., having a diagnosis of depression, mood disorder, anxiety disorder, obsessive–compulsive disorder, schizophrenia, attention deficit disorder, substance use disorder or personality disorder) and/or who were reported to receive or to have received psychiatric treatment (i.e., medication, counselling, psychological intervention) as an indication of experiencing a mental health issue [16]. A recruitment flyer and email were disseminated to mental health practitioners across Australia. Similar recruitment methods were undertaken with mental health and nonprofit organisations that supported carers and families of individuals with mental health issues. The email contained a brief overview about the research, inclusion criteria, and a request for participants. Inclusion criteria specified that adult children needed to: (i) Have/had at least one parent diagnosed with, hospitalised or received treatment for mental health concerns, (ii) have at least one child between 0–18 years of age, and (iii) be residing in Australia. The reason for including a specific age range on adult children’s offspring was to ensure adult children were all parents of dependent children, whose experiences may differ considerably to participants with adult children. For this reason, three participants were excluded from the study leaving a final sample of ten adult children (9 females, 1 male) aged 27–51 years (*M* = 40.30, *SD* = 6.83). Seven participants identified as Caucasian, one participant identified as Aboriginal Australian, and another as Asian. One participant did not specify an ethnicity. Of the ten participants, four reported an annual income of more than $150,000; one participant reported an income between $100,000 and $149,000, three participants reported receiving between $50,000 and $99,999, while two participants chose not to disclose this information. Nine participants reported that their parent had received a formal diagnosis from a mental health practitioner. Seven of the ten participants indicated the presence of past or ongoing mental health concerns themselves with six of those participants having sought professional help. With the exception of one participant, others reported that their symptoms were managed as a result of the professional and informal supports around them. Additional familial information is highlighted in Table 1.

### 2.2. Procedure

The university human research ethics committee provided ethics approval for this research. A semi-structured interview schedule was developed, informed by existing literature in conjunction with the research aim. Participants were asked a series of open-ended questions ranging across childhood, adulthood, and parenthood life stages. Example questions from the interview guide included: ‘how would you describe your childhood experiences in relation to having a parent with mental illness?’ and ‘in what ways, if any, has your childhood experience of parental mental illness impacted your own parenting role?’ Interested participants contacted the primary researcher via email and were then sent an explanatory statement and consent form. Upon receiving written consent, an interview time was arranged. Two individual interviews were conducted in person and eight individual interviews via telephone. All were audio recorded with participant permission, to allow for subsequent transcription. On average, each interview took 80 min to complete and ranged between 58 and 101 min. Member checks were conducted, whereby participants were sent their interview transcript to review, edit, and/or omit any information provided [44]. One participant made additions to clarify the intended meaning of a statement, and the amended transcript was used in the data analysis.

### 2.3. Data Analysis

Interpretative phenomenological analysis (IPA) was deemed an appropriate method for data analysis, given its distinctive epistemological approach [45]. IPA is rooted in describing an individual’s personal perception, emphasises the subjective or ‘human element’ of any given experience [46,47], and is regarded a good fit for analysing lived experiences that are particularly complex and emotionally laden [48]. Although IPA allows for flexibility, there is a core set of processes [49] that are adhered to in interpreting a data set. Interview transcripts were read several times by the primary researcher, who made notes and interpretations with each read. In each round of reading, codes were generated in accordance with the context and interpreted meaning. After this process was completed for each interview, the codes generated were compared across interviews to identify themes relevant to the research aims. Particular emphasis was given to commonalities as well as divergence across interviews. The data analysis process was dynamic in nature, requiring several iterations of reading, codes, and theme generation. The fluid nature of going back and forth in interpreting a data set has been reported as an important process in promoting deeper understanding and authenticity of emergent themes [50]. For each theme, narratives were derived from transcripts to reflect and narrate the essence of the theme through the words of adult children.

## 3. Results

Three superordinate themes were generated in relation to adult children’s own parenthood experiences: ‘this is me’, ‘a whole new world’, and ‘because of you’. The following section unpacks these themes and elaborates on the corresponding subordinate themes using quotes from interviews to ensure their voices are retained.

### 3.1. Superordinate Theme 1: ‘This Is Me’

The first theme, ‘this is me’, is a summation of underlying narratives about adult children wanting to be different from their parents. This theme includes three subordinate themes (see Table 2).

#### 3.1.1. I Got to Do It My Own Way—Being Authentic

Eight of the ten participants described wanting to do things differently from the way their parents parented them.

“I’ve had to be very deliberate about setting my own style. I didn’t want to parent the way that my parents had. So I’ve really had to be deliberate about, you know, how I want it to be, what relationship I want to have with my children and what I want that to look like and I’ve been very deliberate in going about that.”

(Katie)

Although this was the dominant sentiment, three participants acknowledged that there were also positive aspects about parenting they learned from their parents. Several participants recalled that despite the mental illness, their parent made a concerted effort to be hands-on and involved in their lives. Other participants recalled fondly the role their parent played, often describing them as the cornerstone or the glue that kept the family together. For these adult children, reflection was helpful as they consciously identified parenting practices they witnessed in their parents that they wanted to retain, as explained by Jenny, whose Vietnam veteran father was diagnosed with PTSD and bipolar disorder:

“I’ve tried to take what’s good but I’ve also sought out ideas and good ways to do it and sort of done that attachment-based parenting. I haven’t taken this uncritically from my childhood and mirrored it but I have thought about what I liked and my partner’s had some contributions from his family as well.”

(Jenny)

#### 3.1.2. Know You Are Important to Me—Emotional Support for Children

The second subordinate theme contained narratives about the importance of forging a strong emotional connection with their children. Seven participants discussed the importance of creating a home environment that encouraged open communication. For these participants, their goal was to ensure their children knew they could count on them for emotional support. All participants reported that conversations about their parent’s mental illness rarely occurred in their household as they were growing up. Instead, they recalled being left to make sense of the situation on their own or being sent to a grandparent or relative’s house when their parent was having an “episode”, which created further confusion. As a result of their childhood experiences, the emphasis on the ‘soft elements’ of parenting often shone through as being a priority in their own parenting principles.

“We (Tanya and her daughter) can talk about all sorts of things, whether it’s the good things or bad things, having a very open communication, I guess where she sort of feels that if something’s gone wrong or she’s unhappy or any of those sorts of things that we can talk about those things and that I’ll support her and I’ll do what I can to help things get better.”

(Tanya)

#### 3.1.3. Know You Are Important to Me—Promoting Children’s Individuality

Another identified theme was in relation to supporting and fostering children’s individuality. Three participants recalled how their parent with a mental illness exerted immense control over their lives, including what they could wear, the extracurricular activities they participated in, and the friends they had. Accordingly, these participants chose to hide aspects of their life from their parent, with one pretending to like the activities she was forced to be a part of, to avoid confrontations with her mother.

“I learnt that if I was cold towards and distant towards her, it would make her angry, so I would pretend to be what she wanted me to be, so I would pretend that I enjoyed doing the dance and the pageants and all that sort of stuff that she wanted me to do even though I hated it. I would do them because if I didn’t it was worse.”

(Suzanne)

As a result of feeling like they could not be themselves, three participants described the importance of fostering their children’s individuality. Accordingly, they acknowledged that a one-size-fits-all approach to parenting their children was impossible, given children’s inherent individuality, which needed to be celebrated. The following comments by Gabrielle and Suzanne accentuate this point further:

“So for my teenage daughter, if there’s something happening with her friends, okay you can’t change your friends, they’ve sent a nasty text but what’s proactive and positive? And it might simply be having a cup of tea and cleaning out her clothes drawer or doing some math homework. For my son, he’d just want to be able to play Lego, and that’s proactive and positive for him and he, he resolves a lot of conflicts through Lego in his room. And Tessa, she’s the third child, she’s a little hard egg, so I’ll have to work really hard with her to break her down, so a part of my parenting strategy and how its changed is that I don’t apply a one size fits all for them, you know, they’re all different and important to me and that I know their differences [yeah] and then adjust my parenting according to their personalities. That’s really important to me.”

(Gabrielle)

“I try to be as present as possible but I’m also very aware that I don’t own my children, they don’t belong to me, they’re not possessions. They’re very much their own little people, bless them and I know that’s a direct link to my experiences as a child. Like whatever their interests are, whatever they like and whoever their friends are, I couldn’t care less as long as they’re kind, good, honest people and happy.”

(Suzanne)

#### 3.1.4. Call Me Boring If You Must—Prioritising Routines and Stability

The third subordinate theme: ‘call me boring, if you must’ was tied to participants’ reflections about their childhood and the priorities they placed as parents. Adult children noted the unpredictability and uncertainty in the home environment as they were growing up due to parental mental illness, which often was a source of anxiety and fear. Four participants highlighted the importance of creating a stable home environment for their children, as expressively described by Suzanne:

“What I’m very conscious of doing is that I want my kids to know what they’re coming home to every single day. I never want them to feel like they’re coming home from school and… I remember for me, standing at the bottom of the drive way going: “Oh God, what’s happening today?” Like they (children) always know that I pick them up from school, I’m sitting right there, I’m here everyday, we do the same thing everyday. Yes, they get yelled at because they leave their lunchboxes at school or there’s rotting fruit, again! But it’s the same thing, screaming about the apples and it’s the same everyday and as boring as it seems to them sometimes there’s so much safety and stability in that.”

(Suzanne)

### 3.2. Superordinate Theme 2: ‘A Whole New World’

The second theme, ‘a whole new world’, comprised of four subordinate themes (see Table 3) tied to adult children describing parenthood as a role that allowed them to see themselves in a new light.

#### 3.2.1. “Softened as a Person”—Capacity to Love and Be Loved

Parenthood was seen as a steep learning curve but also served as a process of personal recovery for several participants. Anna, in particular, described as a child feeling extremely angry as a result of the turbulent household environment she grew up in due to her father’s bipolar disorder. Unable to withstand the tumultuous home environment, Anna left home as soon as she turned 16 and described becoming “numb” towards her parents and everything she had experienced. Parenthood, however, allowed Anna to see herself in a way she had never seen herself before.

“I just feel like I’ve softened as well as a person. Like I said, I had a very angry childhood, kinda mellowed once I moved out and that’s really where a lot of the processing and finding myself really happened. But I guess it [parenthood] shows me that there is a very strong capacity in me to love and be very attached to someone and I guess that would evoke strength in me.”

(Anna)

#### 3.2.2. My Window to the World—Social Connectedness

The only male participant described parenthood as offering him a way connecting with others:

“Like my son comes and says: “dad, I’ve just joined the museum society. Will you come along with me to the first meeting they’re having next week, with me?” And I said: “yeah I’d love to.” And having done that and been along to the museum society, I’ve made friends there, would you believe? That I would never have met otherwise. That was through him, right? I’ve met other people that I am now friends with, whom I’m learning things from as well. And we’re talking as fathers—father to father—but my son instigated it. So yeah, I’m linking to other groups and sociability.”

(Lionel)

#### 3.2.3. It Takes Two to Tango—Co-Parenting and Complementing Each Other’s Roles

Five participants discussed issues around co-parenting and learning to depend on the other parent. For some, this was challenging, as they had learnt to become very independent as a result of their childhood. For others, it was about making proactive decisions so that their children got to spend quality time with both parents.

“I think also I don’t want to take on too much, like at the moment, me and my husband both work part-time, three days a week and then we share the care of the kids, so that was something that we decided on before we even had kids, that that was going to be our plan because I want them to have a close relationship with their dad because I didn’t get that and I also want us to have work-life balance, so that we’re not too stressed out. So I’m very conscious of not taking on too much.”

(Laura)

#### 3.2.4. “Marching to the Beat I Drum”—Being Kind to Myself

The final subordinate theme acknowledged the need of some to be kind to themselves and care less about societal expectations:

“I think I have the attitude that: “you know what, it’s okay to just do it the way that I think is best.” I think its also as I get older, I feel like its less about other people and more important to march to the beat of your own drums. I think that’s also been incorporated into my parenting mantra… “you know what, if this is just not how most people do it, it’s okay, its working for my family, it works for me.”

(Anna)

### 3.3. Superodinate Theme 3: ‘Because of You’

The final theme, ‘because of you’, consists of reflections by adult children about the parenting challenges they faced as a result of their childhood experiences of parental mental illness (see Table 4).

#### 3.3.1. Whom Do I follow?—Lacking a Reference Point

The first subordinate theme ‘whom do I follow?’ contained feelings of loss and confusion that arose from participants feeling like they lacked a reference point when it came to parenting. Some described this in a positive light, choosing to treat parenthood as a “new career”. However, for four participants, the lack of a parenting reference was anxiety-inducing, as adult children feared not being able to break away from the parenting cycle of how they were raised:

“It’s like I know what should be done but it’s difficult to control myself, I swear. I used to blame it; maybe I still do on my past. Like I cannot break from the patterns that my parents—how my parents raised me.”

(Claudia)

#### 3.3.2. Whom Do I follow?—Difficulty Setting Boundaries

Additionally, the way in which some assumed caring responsibilities at a young age seemed to have a particular impact on some adult children’s parenting styles.

“I have noticed that my experiences from when I was little have come through, in that, like when my sister was four and when mum first got unwell, she (sister) was having really bad tantrums and she’d be really hard to get ready for kinder, and trying to get her dressed in the morning and brush her hair was awful and I just did whatever I could do to make her happy because I didn’t want her to have a tantrum and also I knew that she was much younger than me and it was harder for her to lose mum and I think that comes through now with my daughter sometimes. I would just give in to her own way too easily because I don’t want her to get upset and have a tantrum, so maybe I’m a bit of a pushover sometimes.”

(Laura)

#### 3.3.3. My Childhood vs. Yours—Challenging to Render Emotional Support

Another challenge for some participants was providing emotional support to their children. All participants agreed that the ‘soft elements’ of parenting, such as open communication and emotional support, were important facets in forging strong ties with their children (see Theme 1). However, for two participants, this was occasionally difficult to do because they compared their children’s issues to the ones they faced in their own childhood and in comparison, their children’s troubles seemed trivial.

“I guess it’s all the emotional stuff, you know, like naming feelings and recognising feelings. I mean that’s something that I’ve read multiple times but I still can find it really hard to really kinda sit and go: “ohh okay, so you’re feeling pretty sad about this right now”, I guess because sometimes I feel like I went through really tough times and I can tend to at times get a little bit impatient with people. I feel like: “you don’t even know what tough is” and I kinda have to remind myself that everybody obviously has their tough parts and not to be too impatient but sometimes I do say to my daughter: “really? You don’t realise just how much you actually have… If you had even a tiny bit of what my life was like or what some of their friend’s lives are like…” you know, like you really do have it pretty good.”

(Tanya)

#### 3.3.4. I Just Know Too Much—Experiences Guided Their Choice of Career

Several participants highlighted that their childhood experiences led them into helping professions such as social work, counselling, and child protection services, where they worked closely with vulnerable families. By and large, these adult children acknowledged that their lived experiences helped them in their professional roles, in relating to and building rapport with their clients. The downside, however, was the constant exposure to families where children were maltreated. For some, these experiences made participants more protective of their children, while also making it difficult to trust their children in the care of someone else.

“I think the things I find more challenging are like when she’s away as well, and I wouldn’t say that I have separation anxiety or anything to that extreme but yeah, for me it’s more what other people do with her and what goes on in other people’s houses and those things. I think those have been the hardest for me. I’m not sure if that’s something from mum or even a little bit from work or all of those things combined, but that’s probably been the hardest, yeah. I do get a little bit worried when I probably shouldn’t.”

(Evelyn)

#### 3.3.5. I Wish I Had My Family Tree for Support—Lack of Informal Supports

The final subordinate theme was in relation to the lack of family supports. Most adult children described having positive relationships with their partners, co-workers and friends, whom they turned to for support. However, the biggest challenge was the lack of support from one’s own family. Four participants described how their children had minimal ties with their grandparents. Others indicated that their parent could not be relied upon for childcare duties. Some adult children made a choice to disconnect with their parent with mental illness who was still volatile and, in some cases, verbally abusive towards them and their children; but this distance was not without regret. The missed opportunities to bond over parenthood with their parent along with lost grandparent–grandchild ties were a source of disappointment.

“One of the biggest challenges I’ve had is probably directly related to my childhood is the complete lack of support. I’ve been very much on my own as a parent. My husband and I have been very much on our own. I didn’t have, like my best friend’s just had a baby and she’s very close to her mother and watching her be supported and propped up and having that stuff—I never had any of that. So not having any family support when you have kids is really hard, there’s no break. Even tiny stuff, like having someone to ring and go: “is this normal?” … Because when you’re parenting, none of it is normal, none of it makes any sense and it would have been nice to have someone to call…”

(Suzanne)

## 4. Discussion

The aim of this study was to document adult children’s experiences about their parenting roles in relation to their lived childhood experiences with parental mental illness. Adult children’s perspectives on their parenting experiences and challenges can be used to inform the development of support services. Though different experiences were noted, the overarching sentiment of parenthood served as a salient identity and as a path to personal recovery, notwithstanding certain challenges. The first theme, ‘this is me’, highlighted adult children’s desire to make their own mark when it came to parenting their children. Although some participants highlighted positive elements in their parents’ parenting, which they emulated, for most, the inclination to do things differently or opposite of the parenting they received was salient. A core element within the theme was adult children making an effort to “right the wrong” that had been done to them. For instance, many adult children emphasised the importance of open communication and being emotionally present for their children, which they considered to be lacking in their childhood. They also considered creating routines and a stable home environment important, again because these were missing in their own childhoods. Previous research documented themes such as confusion, unpredictability, and fear [51,52,53] amongst individuals of parents with mental illness. The majority of participants in this study shared comparable narratives but vowed not to raise their children in a similar environment. Hence, participants described how they appreciated the “mundaneness” and routines of everyday life, which afforded adult children and their families an element of comfort and stability.

The second theme, ‘a whole new world’, encapsulated the premise of parenthood being an important part of their identity and personal recovery. Past research has documented the personal recovery process of families of people with severe mental illnesses with four key phases, namely: (i) shock, discovery, and denial, (ii) recognition and acceptance, (iii) coping, and (iv) personal and political advocacy [54]. Based on the family recovery model, the findings here speak to the forging of new ties through new relationships and parenthood as new identities, which are characteristic of the fourth phase, ‘personal and political advocacy’. Through their new relational roles, individuals are able to develop new meanings and perceive themselves in a new light. Some participants acknowledged that becoming a parent created a deep level of appreciation for their own parents. To a certain degree, embarking on a new role offered adult children a different perspective and, in a few cases, aided in relationship reparation. Additionally, the fourth phase is marked by less self-blame and an increased acceptance for things that cannot be changed [54]. This was highlighted by participants as they reflected on the importance of being kind to themselves. Collectively, the theme highlights a myriad of ways in which parenthood has facilitated adult children’s reconciliation and personal recovery process.

Notwithstanding the personal recovery element that parenthood afforded, there appeared to be a unique set of parenting challenges experienced by participants. The final theme, ‘because of you’, is a consolidation of reflections from participants in relation to their parenting struggles. A common theme between the present study and Murphy et al.’s [38] was participants’ concerns about a lack of a parenting reference point or what Murphy called an internal parenting framework. For several participants, the lack of appropriate parenting in their childhood contributed to adult children’s uncertainty and ambiguity in their own parenting roles. Some adult children described this as missing the instinctive element or knee-jerk reaction for being a parent. As a result, adult children went about their parenting role in a more deliberate manner, with some equating it to learning a new skill or starting a new job. Findings from the current study align closely with Murphy and colleagues [38] in that without a reference point, adult children may find it more challenging to set age-appropriate expectations of their children and may be overly critical of themselves in their parental role.

The importance of having a parenting model was evidenced in an earlier study [55], whereby women identified their mothers as the person who most frequently provided them with support. It was found that support from one’s mother increased parental self-efficacy, through vicarious experience and verbal encouragement, highlighting the prominent role of maternal mothers in shaping the next generation’s mothering. However, for participants in the current study, another area of challenge, especially amongst adult children who had no ongoing ties with their parent, was the disappointment over missed opportunities to bond with their parent about parenthood and to share life stories with each other. In conjunction with that was the regret that their children were missing out on grandparent love and connections. Often, adult children wished they had more informal family support to lean on. Further extending the issue regarding support, the current study found that some adult children found it challenging to render emotional support as a result of their childhood experiences. In part, this difficulty arose when adult children compared their children’s problems to the ones they faced as children. The resulting impact of these comparisons made their children’s problems seem trivial in comparison to their own, which caused them to be somewhat impatient and emotionally detached at times.

### 4.1. Implications

Collectively, findings from the current study, along with existing literature, affirm the importance of parenthood, particularly when adequately supported, as a protective element for adult children. Despite the challenges of parenting, adult children acknowledged that parenthood provided them with an opportunity to stay socially connected and provided an added sense of purpose. Nevertheless, there were some clinical implications that need to be duly recognised, if the specific needs of adult children are to be met. Firstly, the lack of a parenting reference point might make it more challenging for these adult children to decipher what normative parental expectations are for children across varying developmental stages. In some instances, expectations were set too high, and in others, too permissive. It is possible that for some adult children, having had to grow up quickly might result in them having the same expectations of their children; while others, in an attempt to create a parent–child relationship high on love and warmth, may end up overcompensating and risk being permissive parents. Given this, it appears that knowing how to strike a balance in parenting might be a necessary area of guidance.

Another avenue of support could be in the form of psychoeducation about developmental stages. Information about developmental milestones could be useful in providing parents with context and tips to manage and help their child during each stage. Additionally, informal supports were generally seen to be more practical and necessary in alleviating the pressures adult children face, and while this may not always be possible through family members, alternative options through which informal supports can be rendered to adult children and their families might be contemplated. For instance, an online support group for adult children could be an effective way to create a supportive network for individuals with shared life experiences. The platform could be an avenue for adult children to ask questions, generate discussions, and render support to each other. Collaboration with religious or community organisations that are able to offer babysitting or childcare services may also be beneficial in allowing parents to schedule in quality couple or personal time, without having to worry about child care duties for several hours. As discussed previously, adult children in this study have shown not only a great level of insight but also openness in seeking professional and informal supports when they felt it was necessary. An external voice from an objective third-party, such as a maternal health nurse, paediatrician or an allied professional, was described as being helpful in reducing self-criticism whilst serving as an element of reassurance. Findings like these are encouraging to the many who work collaboratively with families of people with severe mental illnesses.

### 4.2. Limitations

A number of limitations apply in interpreting the findings. Firstly, the wide variation in participants’ personal experiences of mental illness may have influenced results. For some, their experiences with mental illness may have inadvertently made parenting more challenging, compared to those participants with no history of mental illness. Future studies are needed that compare the narratives of adult children with and without mental illness. Secondly, the voluntary nature of the study may have inadvertently recruited adult children who were coping adaptively or those who had undergone some degree of reflection and reconciliation with their past. Thirdly, more than half of the participants in the present study reported having sought professional help or were themselves employed in a helping profession. Together, these factors could have played a significant role in their ability to adaptively cope with life stressors, including parenting. It should also be cautioned that at least half the participants belonged to the above average SES group based on Australia’s average annual income estimate for 2018 [56]. Hence, findings may differ from adult children in low SES groups, and further research is warranted to investigate this population. Lastly, the majority of participants in this study were mothers and thus, findings may not necessarily translate to fathers in similar situations. While there are common issues shared by mothers and fathers, fathers have their own unique needs distinct from mothers [57], and this area warrants further research. The sample of ten participants provided sufficient data to utilise IPA to examine underlying themes in relation to the research questions. However, as IPA requires the primary researcher to assign meaning and interpretations to the personal accounts of adult children, there is a possibility that interpretations could have been intrinsically influenced by the researcher’s own life experiences and viewpoints [49]. Collectively, the research team involved in collecting and interpreting the data has worked as clinicians and researchers in this field for over twenty years. It is acknowledged that professional experiences may confer credibility but an inevitable part of qualitative research lies in the potential biases of researchers [58]. Nonetheless, to promote rigour, transcripts were sent to participants as a counter-check measure to ensure their narratives were accurately captured. Additionally, codes and themes were derived collaboratively between co-authors and the primary researcher. Future research could interview various stakeholders, such as mental health practitioners and the partner of adult children as a way of obtaining multiple perspectives on the needs and interventions that adult children could benefit from.

## 5. Conclusions

The findings from this study help to advance knowledge in the area of intergenerational parenting in families where a parent has a mental illness. Qualitative studies in this area are vital, as every family who has a person with mental illness has a different story to tell. It is important that these personal narratives are documented so that practitioners can work with existing strengths in adult children and provide further support if warranted. Mental health providers can help to guide adult children of parents with mental illness as they attempt to navigate and make sense of their experiences and the subsequent roles they assume in adulthood. Findings from this study may assist development and refinement of interventions and could provide the groundwork for further research.

## Figures and Tables

**Table 1:** Participant family information.

Pseudonym	Gender	Parent’s Mental Illness/Diagnosis	Participant’s Mental Illness Diagnosis (If Any)	Participant’s Children
Lionel	Male	Father: Depression, anxiety	Diagnosed with depression since age 17 and has ongoing depressive episodes * Has had several visits to psychiatric ward and hospitalisation stays	2 children Ages: 18, 12
Claudia	Female	Mother: Narcissistic personality disorder *(not formally diagnosed but received psychiatric treatment for a brief period)*	None Participant described herself as being very stressed and overly critical of herself *	2 children Ages: 11, 8
Gabrielle	Female	Father: Depression, anxiety, alcohol addiction	Anxiety, post-natal anxiety *	4 children Ages: 16, 10, 8, 4
Anna	Female	Father: Bipolar disorder	Diagnosed with anorexia at age 14 *	2 children Ages: 4, 2
Suzanne	Female	Mother: Depression, narcissistic personality disorder, alcohol addiction. Hospitalised several times for suicidal attempts	Anxiety *	2 children Ages: 12, 4
Laura	Female	Mother: Bipolar disorder Hospitalised several times	None	2 children Ages: 2 and 9 months old
Tanya	Female	Mother: Paranoid schizophrenia, post-natal depression, post-natal psychosis Hospitalised several times	PTSD but has progressively overcome this	5 Children Ages: 4 adult children and a 9-year-old
Jenny	Female	Father: Post-traumatic Stress Disorder (PTSD), bipolar disorder	Transient anxiety due to adjustment issues *	2 children Ages: 12, 9
Katie	Female	Father: Initially diagnosed as mental breakdown but was identified later on as paranoid schizophrenia	None	3 Children Ages: 19, 17, 15
Evelyn	Female	Mother: Bipolar disorder, manic depression, Obsessive Compulsive Disorder (OCD)	None	1 Child Age: 11

* Participant received professional help (e.g., counselling, pharmacology, psychological intervention etc.)

**Table 2:** Superordinate and subordinate themes for: ‘this is me’.

Superordinate Theme	Subordinate Theme	Coding from Transcripts
This is me	I got to do it my own way	Reflective practice (taking the positives but learning from that past and doing this differently) Being authentic Treating parenthood as a career Continuous learning
	Know you are important to me	Emotional support for children Wanting children to know they are prioritised Emphasis on quality parent-child time and interaction Respecting and promoting individuality in children
	Call me boring, if you must	Prioritising routines and stability Emphasis on creating a safe home environment

**Table 3:** Superordinate and subordinate themes for: ‘a whole new world’.

Superordinate Theme	Subordinate Theme	Coding from Transcripts
A whole new world	“Softened as a person”	Parenthood as a reminder of capacity to love and be loved More compassionate as a result of parenthood
	My window to the world	Children keep them connected/social connectedness Helps them stay relevant Worldly and wiser
	It takes two to tango	Learning to share parental responsibilities with partner Learning to co-parent after divorce/separation Making joint parenting decisions Complementing each other’s roles
	“Marching to the beat I drum”	Being kind to myself Learning to care less about societal expectations No perfect parent, just a real one Parenthood as a salient identity and protective factor

**Table 4:** Superordinate and subordinate themes for: ‘because of you’.

Superordinate Theme	Subordinate Theme	Coding from Transcripts
Because of you	Whom do I follow?	Lacking a parenting reference point resulted in parenting extremes Tendency to engage in permissive parenting and difficulties setting boundaries Feeling like they are never good enough as parents
	My childhood vs. yours	Tendency to compare children’s childhood to their own Found it challenging to render emotional support and/or relate to children’s problems
	I just know too much	Lived experience guided career choice Lived experience and professional experience made it difficult for participants to trust children in the care of someone else Feeling constantly anxious
	I wish I had my family tree for support	Biggest challenge was the lack or absence of informal support (e.g., grandparents, relatives), even though support was available for some through partner and colleagues Missing grandparent connection and ties Less ‘me’ and ‘us’ time as a result of reduced informal supports
